# Taxogenomics and Systematics of the Genus *Pantoea*

**DOI:** 10.3389/fmicb.2019.02463

**Published:** 2019-10-30

**Authors:** James T. Tambong

**Affiliations:** Ottawa Research and Development Centre, Agriculture and Agri-Food Canada, Ottawa, ON, Canada

**Keywords:** phylogenomics, taxonomy, systematics, average nucleotide identity, tetranucleotides, codon usage

## Abstract

Members of the genus *Pantoea* are Gram-negative bacteria isolated from various environments. Taxonomic affiliation based on multilocus sequence analysis (MLSA) is used routinely for inferring accurate phylogeny and identification of bacterial species and genera. Partial sequences of five housekeeping genes (*fus*A, *gyr*B, *leu*S, *rpo*B, and *pyr*G) were extracted from 206 draft or complete genomes of *Pantoea* strains publicly available in databases and analyzed together with the representative sequences of the 25 validly published *Pantoea* type strains to verify and assess their phylogenetic assignations. Of a total of 159 strains assigned to species level, 11.3% of the non-type strains were incorrectly assigned within suitable *Pantoea* species. The highest proportion of misidentified strains was recorded in *Pantoea vagans*, 8 out of 15 (53.3%) inaccurate assignations at the species level. One probable reason for this incorrect classification could be the method previously used for strain identification. Forty-seven (22.8%) genome sequences were from strains identified at the genus level only (*Pantoea* sp.). A combination of MLSA, average nucleotide identities [ANI and MuMmer-based ANI (ANIm)], tetranucleotide usage pattern (TETRA), and genome-based DNA-DNA hybridization (gDDH) data was used to accurately assign 25 of the 47 strains to validly published *Pantoea* species, while 17 strains could be assigned as putative novel species within the genus *Pantoea*. Four genomes designed as *Pantoea* sp. were identified as *Mixta calida*. Positive and significant correlation coefficients were computed between MLSA and all the indices derived from whole-genome sequences being proposed for species delimitation. gDDH exhibited the best correlation with MLSA while TETRA was the worst. Accurate species-level identification is key to a better understanding of bacterial diversity and evolution. The MLSA scheme used here could be instrumental to determine the correct taxonomic status of new whole-genome sequenced *Pantoea* strains, especially non-type strains, before depositing into public databases.

## Introduction

Members of the genus *Pantoea* are non-encapsulated, non-spore-forming Gram-negative bacteria of the Enterobacteriaceae family. The genus consists of 25 described species and two subspecies^1^ isolated from various environments such as water, soil, human, animals, and plants (Deletoile et al., 2009; Tambong et al., 2014). However, seven of the 25 species have recently been classified into two new genera. Previously, *P. citrea*, *P. terrea*, and *P. punctata* were transferred to the genus *Tatumella* (Brady et al., 2010). Recently, *Pantoea calida*, *Pantoea gaviniae*, *Pantoea theicola*, and *Pantoea intestinalis* were moved to a new genus *Mixta* (Palmer et al., 2018). This suggests that the taxonomy of the genus is evolving probably due to the use of improved taxonomic methodologies. Furthermore, it is an indication that biochemical or nutritional characteristics previously used to differentiate *Pantoea* species/strains were inadequate.

The use of 16S rRNA is an essential tool for the classification and systematics of members of the genus *Pantoea*. However, 16S rRNA gene sequences show low resolution at the intrageneric level (Mulet et al., 2010; Gonzalez et al., 2013), making reliable species- and subspecies-level identifications not possible. Currently, bacterial species delineation is based on multilocus sequence analysis (MLSA) of marker genes such as 16S rRNA, 23S rRNA, *rpo*B, *gyr*B, and *dna*K (Konstantinidis and Tiedje, 2007; Liu et al., 2012; Paul et al., 2019). Housekeeping genes such as *leu*S, *fus*A, *gyr*B, *rpo*B, *rlp*B, *inf*B, and *atp*D have been used routinely to refine interspecific phylogenetic positions of species from the genus *Pantoea* (Brady et al., 2008; Deletoile et al., 2009; Tambong et al., 2014; Palmer et al., 2017). The MLSA approach based on six genes (*leu*S, *fus*A, *gyr*B, *rpo*B, and *rlp*B) was found to provide a more robust and reliable DNA relatedness and species delineation in *Pantoea* (Tambong et al., 2014). Also, comparative analysis of the single gene topologies to that derived from concatenated data identified *leu*S as a reliable phylogenetic marker for the genus *Pantoea* (Tambong et al., 2014).

Wet-lab DNA–DNA hybridization (wDDH) technique has been the gold standard for inferring genomic similarity between two strains for classification purposes (Goris et al., 2007; Rosselló-Móra et al., 2011). However, the approach has inherent drawbacks such as irreproducibility between laboratories, high error and failure to produce accumulative databases (Sneath, 1989; Stackebrandt, 2003) with requests to replace wDDH methodologies with reliable techniques (Stackebrandt et al., 2002; Goris et al., 2007; Rosselló-Móra et al., 2011).

Whole-genome sequencing provides complete and draft chromosome data that can be used to better understand the evolutionary and taxonomic relationships in bacteria in general (Coenye et al., 2005; Mulet et al., 2010; Thompson et al., 2013) and members of the genus *Pantoea*, in particular. The use of genome-based phylogeny is improving bacterial taxonomy leading to a substantial revision on the tree of life (Parks et al., 2018). Taxogenomics of bacteria could be defined as a cohesive comparative genomics approach that combines MLSA, average nucleotide identity (ANI), codon usage bias, core, and pan-genome analysis as well as supertree analysis and other genomic signatures (Thompson et al., 2013). With advances in whole genome sequencing (wgs) and bioinformatics tool developments, these genome-based methods are fast replacing the wDDH techniques in classification of prokaryotes. These genome-based methods provide a more reproducible taxonomic system as well as creating accumulative databases. The methods used in our study of the genus *Pantoea* include genome-to-genome distance (GGDC; Meier-Kolthoff et al., 2013); MuMmer-based average nucleotide identity (ANIm; Goris et al., 2007); (ANI; Jain et al., 2018); tetra usage patterns (TETRA; Teeling et al., 2004); and codon usage (Duret, 2002).

Genome sequencing and analysis of strains will remain key tools in improving our understanding of the taxonomy of prokaryotes. There are over 222,000 publicly available bacterial genomes based on the PATRIC (Wattam et al., 2014; accessed in March 22, 2019). It is expected that the number of genomes will grow exponentially as improved wgs techniques and bioinformatics tools are developed, indicating that genome data will influence the classification and systematics of bacteria for years to come. As such, assigning the genomes of new and old strains to the correct and authenticated bacterial species is primordial, giving that genome data analysis is becoming the “new” gold standard.

There are 253 whole genome sequences of *Pantoea* in the NCBI database (accessed on March 22, 2019). There is no report on phylogenomic studies of majority of the *Pantoea* genomes in GenBank. Palmer et al. (2017) reported a phylogenomic study of 24 *Pantoea* genomes in a comparative study with selected members of the *Erwinia* and *Tatumella* genera. A preliminary MLSA study (data not shown) of six genes (*fus*A, *gyr*B, *leu*S, *pyr*G, *rpo*B, and *rlp*B) derived from publicly available *P. ananatis* genomes indicated potential incorrect species-level placements. This observation prompted the analysis of the majority of the *Pantoea* genomes available in NCBI database. The objectives of this study were: (1) to determine the taxonomic affiliation of the 230 whole genome sequences publicly available in the NCBI database; (2) to compare *leu*S and MLSA with the genome-based methods for species-level delineation; and (3) to perform a comparative study of the genome-based methods. The *leu*S gene is targeted because it is reported to be a reliable phylogenetic marker for the genus *Pantoea* (Tambong et al., 2014). Its potential correlation to genome-based methods would strengthen its use as a reliable “first-aid” tool for preliminary species-level determination within the genus *Pantoea*.

## Materials and Methods

### Genome Downloads and Sequencing

Whole genome sequence (wgs) data of 234 *Pantoea* genomes were downloaded from GenBank at NCBI^2^ using the getgbk.pl script as implemented in CMG-Biotools (Vesth et al., 2013). Genome sequences were extracted from GenBank files and saved in FASTA format using the saco_convert script (Jensen and Knudsen, 2000). The downloaded genomes were scanned using RNAmmer (Lagesen et al., 2007) or BLASTn (Altschul et al., 1990) for the presence of 16S rRNA or *leu*S gene. Genome sequences that do not possess the 16S rRNA and *leu*S genes were excluded. Based on this criterion, 28 genome sequences mainly from the Uncultivated Bacteria and Archaea (UBA) data set (Parks et al., 2018) were not included in the analysis. Also, *Candidatus Pantoea carbekii* strains were excluded due to the small genome size (<1.9 Mb). A total of 206 NCBI genomes were used in this study (Supplementary Table S1).

In addition, three *de novo* genomes of *P. eucalypti* LMG 24197_T_, *P. anthophila* LMG 2558_T_, and *P. deleyi* LMG 24200_T_ were sequenced. The genomes of *P. eucalypti* and *P. anthophila* were sequenced since there were no representative type strains or reference strains in publicly available databases. A new genome sequence of *P. deleyi* LMG 24200_T_ was generated because the available Genbank entry (MIPO00000000) has a high number of contigs (316) (Supplementary Table S1). Reference strains were selected based on preliminary MLSA BLASTn that resulted in a 99–100% similarity with the corresponding type strain, for example *P. vagans*. The draft genomes of these three type strains were determined by the Génome-Québec Innovation Centre (Montreal, QC, Canada) using Illumina MiSeq paired-end sequencing technology; and the raw reads assembled using ABySS version 1.5.4 (Simpson et al., 2009) as previously described (Adam et al., 2014; Tambong et al., 2016). The generated genome sequences were annotated using PATRIC (Wattam et al., 2014) and RAST (Aziz et al., 2008) and deposited in GenBank with accession numbers VHJB00000000, VHIZ00000000, and VHJA00000000 for *P. eucalypti* LMG 24197_T_, *P. anthophila* LMG 2558_T_, and *P. deleyi* LMG 24200_T_ respectively.

### Multilocus Sequence Analysis

Partial sequences of *fus*A, *gyr*B, *leu*S, *pyr*G, and *rpo*B, previously used to infer phylogenetic relatedness of *Pantoea* species (Tambong et al., 2014), were extracted from each genome used in this study. The corresponding gene sequences of the type strains of *Pantoea* species reported in previous studies and available in NCBI were retrieved. The genomes of type strains of newly validly published *Pantoea* species were used to obtain the partial sequences of the required gene loci. For MLSA, BLASTn and phylogenetic analyses, the genes were concatenated (total length, 2648 nt) in the following order: *fus*A (588 nt), *gyr*B (722 nt), *leu*S (623 nt), *pyr*G (306 nt), and *rpo*B (409 nt). A customized database of concatenated sequences was generated using the NCBI makeblastdb command and Blastn performed as previously reported (Tambong, 2017). The concatenated nucleotide sequences were aligned using the very accurate criterion of the CLC Genomics Workbench version 12.0 (CLC-GW12) alignment module with gap open cost of 10.0 and gap extension cost of 1.0. Aligned concatenated nucleotide sequences were used to infer maximum-likelihood (ML) phylogenies using PhyML version 3.0 (Guindon et al., 2010) and CLC-GW12 phylogenetic tree reconstruction module. The best substitution model was the general time reversible (GTR) with rate variation (G) and topology variation (T), selected on the basis of the lowest values of Bayesian information criterion (BIC = 42,167.97), Akaike information criterion (AIC = 41,607.69), and Akaike corrected information criterion (AICc = 41,614.72). ML phylogenies were executed with subtree pruning and regrafting (SPR) with nearest-neighbor interchange (NNI) tree improvement algorithms with 1000 bootstrap replicates. Trees were visualized as circular phylogram with bootstrap values above 70% showed at the group or subgrouping branching nodes.

### Comparison of Whole-Genomes

Genome sequence-based parameters used to compare the strains include genome-to-genome distance calculator (gDDH), TETRA, ANI, and MuMmer-based ANI (ANIm). The gDDH tool is based on the principle of genome blast distance phylogeny (GBDP) implemented in two phases (Meier-Kolthoff et al., 2013). The first phase of the GGDC function (Bray et al., 2003) is a local alignment by BLAST to identify intergenomic matches referred to as high-scoring segment pairs (HSPs) between the two genome sequences. In the second step, these HSPs are converted to a distance value d (X, Y) using a specific distance function with a species cut-off value of 70% similarity. The GGDC data were computed using the web-based tool hosted at http://ggdc.dsmz.de (Meier-Kolthoff et al., 2013). The most recently updated version, GGDC 2.1, was used in this study. This version has improved prediction models as well as confidence-interval estimation. The statistical inferences of the TETRA and the ANIm values were done using a standalone JSpecies software downloaded from http://www.imedea.uib.es/jspecies. The ANIm index was calculated based on the MUMmer ultra-rapid aligning tool (Kurtz et al., 2004). Species cut-off value for ANIm was 96% and >0.99 for the TETRA signatures (Richter and Rossello-Mora, 2009). ANI values were computed using the FastANI algorithm (Jain et al., 2018), a newly published method using alignment-free approximate sequence mapping. The FastANI tool fragments a given query genome into overlapping fragments of a specific size. The sized fragments were then mapped to the reference genome using Mashmap (Jain et al., 2018). The target range of ANI estimate is 80–100% (Jain et al., 2018). In the current study, a stringent cut-off threshold of 96% was implemented. Also, clustering analysis of codon usage data and visualization by heatmap was performed using CMG-Biotools pipeline (Vesth et al., 2013) on genomes that exhibited gDDH, ANI, ANIm, and TETRA values that are below the cut-off values for species delineation with respect to the type/reference strain of the affiliated *Pantoea* species. The codon usage data was calculated using BioPerl modules (Stajich et al., 2002), and the plots were produced using Perl and Gnuplot as implemented in CMG-Biotools pipeline (Vesth et al., 2013).

### Correlation Between *leu*S, MLSA, and Genome-Based Indices

Pairwise parametric and non-parametric correlation analyses were computed between all data of *leu*S, MLSA, and genome-based indices (gDDH, ANI, ANIm, and TETRA) as previously reported (Gomila et al., 2015). Pearson’s parametric correlations and Spearman’s or Kendall tau rank non-parametric correlation coefficients were calculated using the *cor* function (Langfelder and Horvath, 2012) as implemented in R-statistics (R Core Team, 2014). To visualize the correlation matrix, heatmaps were generated using the *corrplot* function of R-statistics.

## Results

### Summary Statistics and General Features of the Genomes Used in This Study

*De novo* assembly of the genomes of *P. anthophila* LMG 2558_T_ (VHIZ00000000), *P. deleyi* LMG 24200_T_ (VHJA00000000), and *P. eucalypti* 24197_T_ (VHJB00000000) have 66, 86, and 104 contigs (with sizes ranging 4.7–4.8 Mb), respectively. The wgs of the three genomes had 4628, 4599, and 4760 coding sequences, respectively. Two hundred and six complete and draft wgs were downloaded from NCBI GenBank (Supplementary Table S1). These include genome sequence entries classified as *P. ananatis* (56), *P. agglomerans* (33), *P. vagans* (15), *P. stewartii* (11), *P. dispersa* (12), *P. allii* (5), *P. rodasii* (3), *P. eucrina* (2), *P. brenneri* (2), *P. rwandensis* (2), while all the other species had a single genome sequence (Supplementary Table S1). Forty-seven entries were taxonomically assigned to *Pantoea* sp. The majority (91.3%) of the sequences had a genome size between 4.3 and 5.9 Mb (Supplementary Table S1). *Pantoea* sp. At-9b and *Pantoea cypripedii* LMG 2657_T_ had genome sizes of 6.3 Mb and 6.6 Mb, respectively. Two strains, PaVv7 (CEW00000000) and PaVv9 (CEGN00000000), currently affiliated to *P. vagans*, had genome sizes of 9.75 Mb, significantly higher than the expected values. Fourteen genome sequences had a size between 2.3 and 3.99 Mb, most of which are from the UBA project.

**FIGURE 1:**
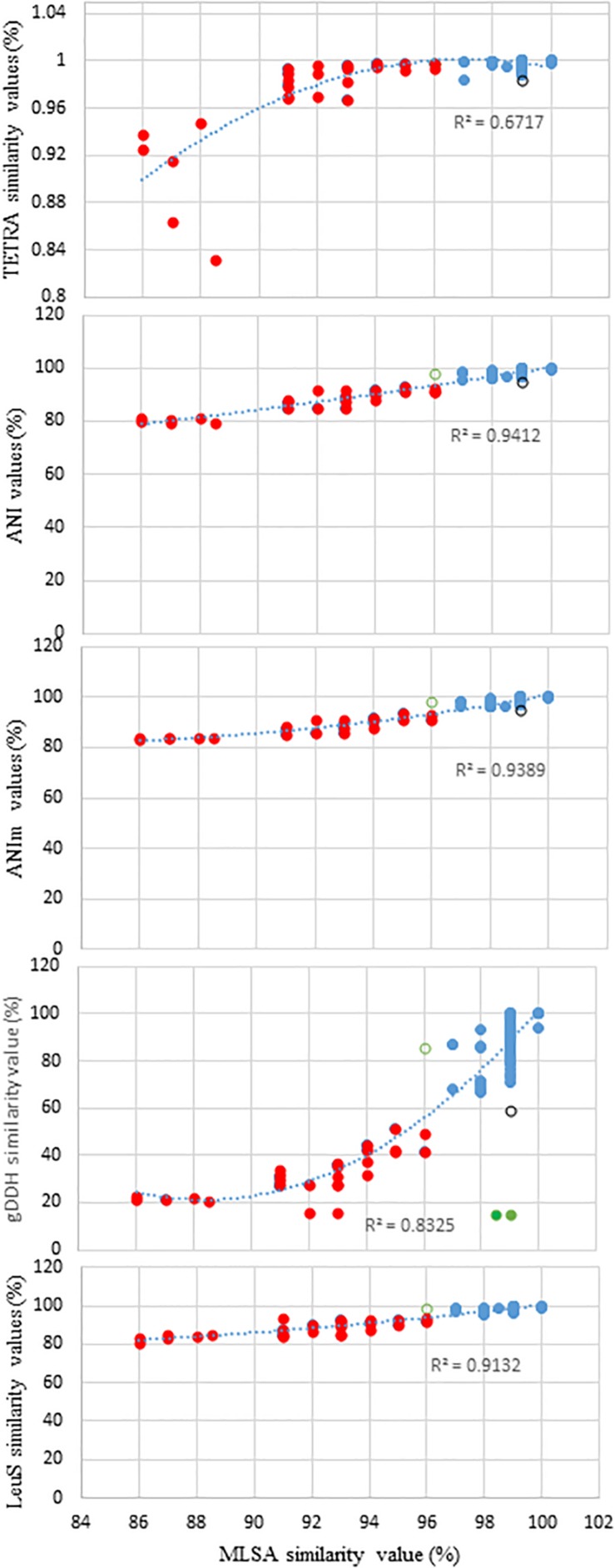
Distribution of the average nucleotide identities (ANI), MuMmer-based average nucleotide identity (ANIm), tetranucleotide usage pattern (TETRA), *leu*S, and genome-based DNA-DNA hybridization (gDDH) data of all the genome sequences plotted against MLSA with a species delimitation cut-off value of 97%. Blue dots, accurately identified to the species-level: gDDH>70%; ANI or ANIm>96%; and TETRA>0.998; red dots, indices lower than the respective cut-off threshold values (misidentified strains and potential novel species); and green dots, two genome sequences (CEGN00000000 and CEMW00000000) with MLSA>97%, ANI or ANIm >96% but gDDH values <70%, potentially due to duplicated gene copies. Black circle depicts a genome sequence with MLSA values of 99% while ANI, ANim, TETRA, and gDDH values are below the cut-off threshold for species delineation. Green open circle shows a single genome with MLSA values of 96.0% (below the threshold of 97%) while the other indices exhibited values higher than their respective cut-off threshold.

Figure 1 shows the distribution of ANI, ANIm, TETRA, and gDDH data of the genome sequences plotted against MLSA with a species delimitation cut-off value of 97%. The genome entries depicted as blue dots were accurately identified to the species-level based on the species delineation threshold of each of the indices: gDDH≥70%; ANI or ANIm≥96%; and TETRA≥0.998 (Figure 1). Two *Pantoea* genome entries (green dots) had MLSA≥98%, ANI or ANIm≥96% but gDDH values<70% (Figure 1). In contrast, the genome entries showed as red dots had indices lower than the respective cut-off threshold values. This clustered all the misidentified strains as well as potential novel species. A genome entry (black circle) had MLSA value of 99% while all the other indices were below their respective cut-off threshold for species delineation. Also, a single genome (green open circle) had MLSA value of 96.0% (below the threshold of 97%) while the other indices were higher than their respective cut-off threshold (Figure 1). The relationship between MLSA and the other indices is non-linear with highly significant coefficients of determination (Figure 1).

### Species-Level Verification of Strain Identity

Species-level identity of strains within a given *Pantoea* species was verified using *leu*S, MLSA, ANI, ANIm, and gDDH data in a comparative analysis with the corresponding type or reference strain. Fifty-four of the 56 *P. ananatis* genome entries showed values of the indices above or equal to the cut-off threshold values while two had pairwise TETRA, ANI, ANIm, and gDDH values lower than the expected value for species affiliation. Two strains, MHSD5 (PUEK00000000) and MR5 (LBFU00000000) exhibited values that are below the cut-off levels to be affiliated with *P. ananatis* species (Table 1), an indication of incorrect species-level assignation. This discrepancy is also evident on the dendrogram generated using the PermutMatrix software (data not shown). Strain MHSD5 showed values above cut-off values for all parameters when compared to the type strain of *P. eucalypti* while strain MR5 (LBFU00000000) showed high taxonomic association with *P. stewartii* subsp. *stewartii* (Table 1).

**TABLE 1:** Proposed taxonomic affiliations based on concatenated MLSA, *leu*S, and whole-genome indices of genome sequences incorrectly assigned at the species level^1^.

**Previous classification**	**Type strain/strain**	**Genome**	**Proposed**	**MLSA (%)**	***leu*S (%)**	**gDDH (%)**	**ANI (%)**	**ANIm (%)**	**TETRA**
	**code**	**accession#**	**classification^2^**						
*Pantoea ananatis*	LMG 2665^T^	JFZU00000000							
	MHSD5	PUEK00000000	*P. eucalypti*	99.00	99.00	93.70	99.19	99.23	0.9998
	MR5^3^	LBFU00000000	*P. stewartii* subsp. *stewartii*	99.00	99.00	92.30	98.58	99.13	0.9927
*Pantoea agglomerans*	DSM 3493^T^	FYAZ00000000							
	299R	ANKX00000000	*P. eucalypti*	99.00	99.00	93.00	98.77	99.10	0.9881
	FDAARGOS_407	PDEG00000000	*P. vagans*	98.00	98.00	68.10	96.05	96.22	0.9969
	NFPP29	FUWI00000000	*P. eucalypti*	99.00	98.00	94.30	99.19	99.28	0.9994
*Pantoea vagans*	C9-1	CP002206							
	ND02	CP011427	*Pantoea* sp. nov.	91.00	88.00	30.70	87.06	86.93	0.9939
	FBS135	CP020820	*P. eucalypti*	99.00	99.00	94.60	99.26	99.31	0.9992
	848_PVAG	JUQR00000000	*P. septica*	97.00	97.00	68.20	95.70	96.38	0.9834
	ZBG6	LFQL00000000	*P. agglomerans*	99.00	99.00	88.60	98.38	98.62	0.9994
	Pa	MUJJ00000000	*P. agglomerans*	99.00	98.00	79.20	97.31	97.69	0.9997
	PaVv11	CEFP00000000	*P. agglomerans*	99.00	99.00	88.20	98.42	98.64	0.9998
	PaVv7	CEMW00000000^3,4^	*P. agglomerans*	99.00	99.00	15.30	96.83	96.85	0.9962
	PaVv9	CEGN00000000^3,4^	*P. agglomerans*	98.50	99.00	15.30	96.55	96.59	0.9945
*Pantoea allii*	LMG 24248^T^	NTMH00000000							
	PNG 92-11	QGHE00000000	*P. agglomerans*	99.00	98.00	79.00	97.28	97.66	0.9992
	PNA 02-18	RBXY00000000	*Pantoea ananatis*	99.00	99.00	92.20	99.19	99.33	0.9911
*Pantoea eucrina*	LMG 5346^T^	MIPP00000000							
	Russ	MAYN00000000	*Pantoea* sp. nov.	95.00	93.00	51.70	93.30	93.43	0.9972
*Pantoea rwandensis*	LMG 26275^T^	MLFR00000000							
	ND04	CP009454	*Pantoea* sp. nov.	94.00	93.00	37.20	89.89	89.53	0.9950
*Pantoea rodasii*	LMG 26273^T^	MLFP00000000							
	ND03	JTJJ00000000	*Pantoea* sp. nov.	91.00	88.00	29.70	85.96	86.42	0.9936

*^1^MLSA, MultiLocus Sequence Analysis (species cut-off = 97%) was done by concatenation of *fus*A, *gyr*B, *leu*S, *pyr*G, and *rpo*B except for strains PaVv7 and PaVv9 which did not have *fus*A. Genome-based DNA-DNA hybridization (gDDH; species cut-off = 70%); ANI, average nucleotide identity (species cut-off = 96%); ANIm, MuMmer-based ANI (cut-off = 96%) and TETRA, Tetranucleotide values (species cut-off = 0.998). ^2^Other NCBI genome accession numbers of reference or type strains used to propose new taxonomic affiliations are CP017581, MLJJ00000000, and VHJB00000000 (this study) for *P. stewartii* subsp. *stewartii, P. septica*, and *P. eucalypti*, respectively. ^3^Genome sequences with no or fragmented *fus*A gene fragment and as such MLSA data is based on four genes. ^4^Strains PaVv7 and PaVv9 had two identical MLSA gene copies but only single copies were used in analysis. This might have affected the gDDH values.*

**FIGURE 2:**
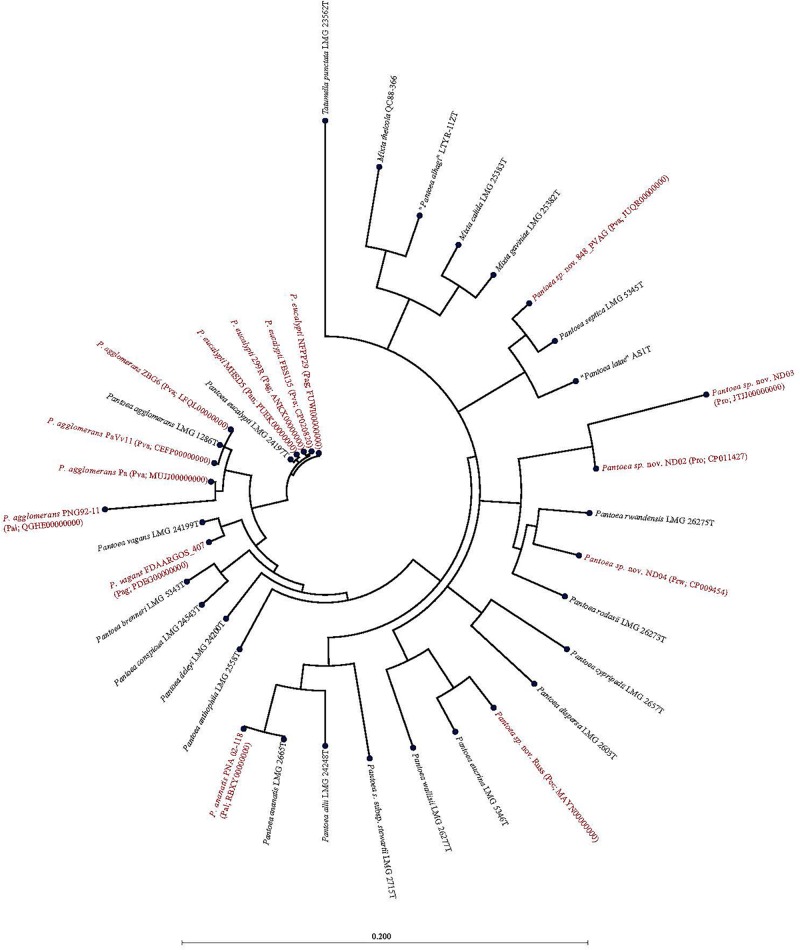
Maximum likelihood phylogenetic analysis of complete and draft genomes (red) with incorrect species-level assignment relative to validly described type strains (black) of the genus *Pantoea*. For the incorrectly assigned genomes (red) in bracket: previous incorrectly assigned species (Pag, *Pantoea agglomerans*; Pal, *P. allii*; Pan, *P. ananatis*; Pec, *P. eucrina*; Pro, *P. rodasii*; Prw, *P. rwandensis*; and Pva, *P. vagans*). Strains MR5, PaVv7, and PaVv9 were not included in the phylogenetic analysis because the draft genomes had short and fragmented *fus*A gene length sequence.

Of the 15 strains assigned to *P. vagans*, eight exhibited values below the species level cut-off threshold of each of the parameters (Table 1). Eight of 15 *P. vagans* genomes had values of gDDH<70%, ANI or ANIm<96% and TETRA<0.998 as well as MLSA<97%, an indication that these genomes do not belong to this species. This incongruity is also evident by two distinct clusters on the dendrogram generated using PermutMatrix (Supplementary Figure S1). Six strains (ZBG6, LFQL00000000; Pa, MUJJ00000000; PaVv11, CEFP00000000; PaVv7, CEMW000000004; and PaVv9, CEGN000000004) exhibited high taxonomic associations instead with *P. agglomerans* (Table 1). Even though strains PaVv7 and PaVv9 had gDDH values below the cut-off threshold of 70%, *leu*S and MLSA data were used to confirm the taxonomic affiliation to be *P. agglomerans*. One strain, FBS135 (CP020820) was correctly assigned to *P. eucalypti* (Table 1). These associations were highly corroborated by phylogenetic analysis. Figure 2 shows the correct phylogenetic affiliations of the previously misidentified complete or draft genome sequences. Strain ND02 (CP011427) exhibited values below the species cut-off threshold of all parameters (*leu*S, 88.0%; MLSA, 91.0%; gDDH, 30.7%; ANI, 87.1; ANIm, 86.9; TETRA, 0.9939) with the closest species being *P. rodasii* (Table 1 and Figure 2). Strain 848 (JUQR00000000) exhibited borderline DNA relatedness with *P. septica* as the closest species (Table 1 and Figure 2).

Of the 33 strains reported to be *P. agglomerans*, three strains, 299R (ANKX00000000), NFPP29 (FUWI00000000) and FDAARGOS_407 (PDEG00000000), showed low taxonomic relatedness to this species (Table 1). Strains 299R and NFPP29 instead showed high genomic similarity with the type strain of *P. eucalypti* (Table 1 and Figure 2). However, strain FDAARGOS_407 showed borderline values below or above cut-off threshold for all computed parameters (*leu*S, 98%; MLSA, 98%; gDDH, 68.1%; ANI, 96.01%; ANIm, 96.2%; TETRA, 0.9969) with *P. vagans* as the closest species (Table 1 and Figure 2). With respect to genome sequences affiliated with the species *P. allii*, two strains PNG 92-11 (QGHE00000000) and PNA 02-18 (RBXY00000000) showed high relatedness instead with *P. agglomerans* and *P. ananatis*, respectively (Table 1 and Figure 2). The single strains MAYN00000000, CP009454, and JTJJ00000000, previously assigned to *P. eucrina*, *P. rwandensis*, and *P. rodasii*, respectively, showed low relatedness to the corresponding type strain (Table 1) but remained one of the two closest species.

### Species-Level Classification of Entries Classified as *Pantoea* sp.

**FIGURE 3:**
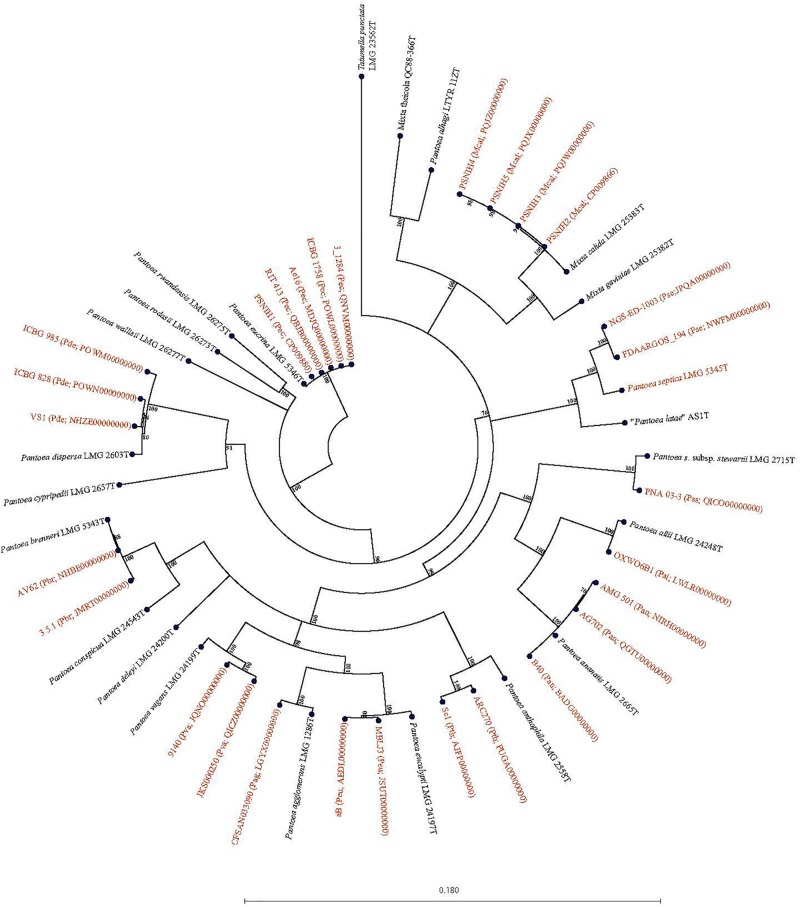
Maximum Likelihood phylogenetic tree of five concatenated genes of 29 complete and draft genomes previously reported as *Pantoea Mixta* sp. showing species level taxonomic affiliations to validly described type strains of *Pantoea* and *Mixta* species. The type strain of *Pantoea*, and *Mixta* species associated with a given *Pantoea* sp. genome entry based on the collective data of the six parameters (*leu*S, MLSA, gDDH, ANI, ANIm, and TETRA) recorded is given in brackets before the NCBI genome accession number: Pag, *P. agglomerans*; Pal, *P. allii*; Pan, *P. ananatis*; Pth, *P. anthophila*; Pbr, *P. brenneri*; Pde, *P. dispersa*; Peu, *P. eucalypti*; Pec, *P. eucrina*; Pse, *P. septica*; Pss, *P. stewartii* subsp. *stewartii*; aPva, *P. vagans; Mcal; Mixta calida*. *Pantoea*, *Mixta*, and *Tatumella* type strains are in black.

**FIGURE 4:**
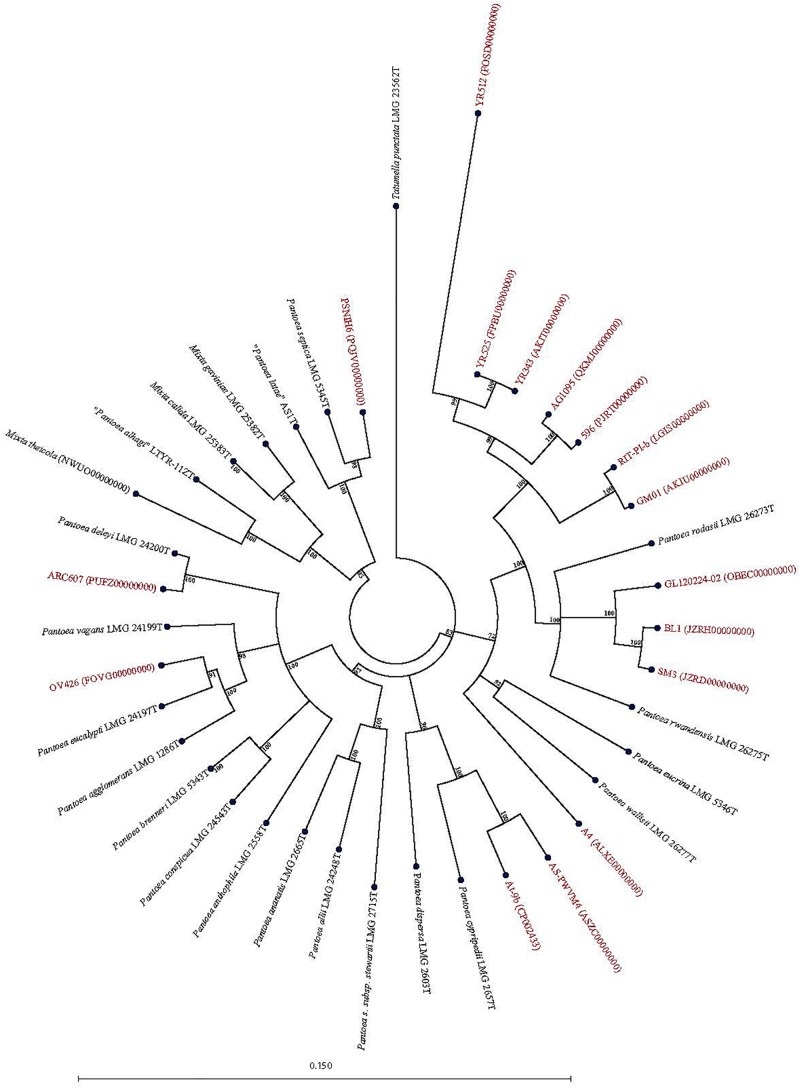
Maximum Likelihood phylogenetic tree of potential novel *Pantoea* species (red) previously reported as *Pantoea* sp. in NCBI database in phylogenetic association with *Pantoea, Mixta, and Tatumella* type strains (black).

Forty-seven *Pantoea* genomes reported as *Pantoea* sp. were analyzed using *leu*S, MLSA, gDDH, ANI, ANIm, and TETRA data to determine their taxonomic similarities/relatedness relative to known and validly published *Pantoea* species. Supplementary Table S2 shows the proposed species-level taxonomic affiliations based on *leu*S, concatenated MLSA and whole-genome analyses of strains previously reported as *Pantoea* sp. Twenty-five of the 47 genomes could be reliably assigned to 11 validly described species based on high taxonomic relatedness as indicated by indices significantly above the cut-off threshold of the different parameters (Figure 3 and Supplementary Table S2). Six strains (3_1284, QNVM00000000; Ae16, MDJQ00000000; RIT 413, QBJB00000000; PSNIH1 CP009880; BRM17, PEFU00000000; ICBG 1758, POWL00000000) were highly taxonomically similar to the type strain of *P. eucrina* (Figure 3). Three strains could be reliably affiliated with *P. dispersa* while two genomes each previously assigned as *Pantoea* sp. taxonomically were affiliated with *P. anthophila*, *P. eucalypti*, *P. ananatis*, *P. septica, P. brenneri*, and *P. vagans* (Figure 3 and Supplementary Table S2). In addition, strains CFSAN033090 (LGYX00000000), OXW06B1 (LWLR00000000), and PNA03-3 (QICO00000000) showed high taxonomic relatedness with *P. agglomerans*, *P. allii*, and *P. stewartii* subsp. *Stewartii*, respectively (Figure 3 and Supplementary Table S2). Four of the strains (PSNIH2, CP009866; PSNIH3, PQJW00000000; PSNIH5, PQJX00000000; PSNIH4, MRBS00000000) had high taxonomic similarities with *Mixta calida* (previously *Pantoea calida*) (Figure 3 and Supplementary Table S2). Seventeen genome sequences could not be assigned to a valid taxonomic *Pantoea* species and the closest type strains were: *P. rodasii* (Nine strains), *P. septica* (Two strains), and *P. cypripedii* (Two strains) while *P. anthophila*, *P. deleyi*, and *P. rwandensis* had one strain each (Figure 4 and Supplementary Table S2). These strains could be potential novel species. Strain 1.19 (MRBS00000000) showed values lower than the cut-off thresholds and the closest species was *Mixta gaviniae*, previously *Pantoea gaviniae* (Supplementary Table S2).

### Correlation Between MLSA, *leu*S, and Whole-Genome-Based Indices

**FIGURE 5:**
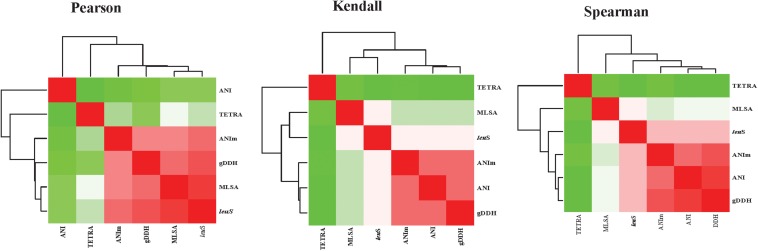
Heatmaps of the correlation coefficients between the different parameters [leuS, Multilocus sequence analysis (MLSA), Average nucleotide Identity (ANI), MuMmer-based average nucleotide identity (ANIm), and genome-based DNA-DNA hybridization (gDDH)]. Correlation analyses were performed using the *cor* function and visualized using *corrplot* as implemented in R-Statistics (R Core Team, 2014).

Average nucleotide identities (ANI), ANIm, TETRA, gDDH, MLSA, and *leu*S comparisons were computed for 206 genomes, generating a total of 1254 data points. Correlation analyses between all the parameters were performed (Figure 5 and Supplementary Table S3). All correlation coefficients were significant at *p* = 0.00. High correlations were found between MLSA and *leu*S (0.923 Pearson’s coefficient, 0.790 Spearman’s rho, and 0.728 Kendall’s tau) and between MLSA and gDDH (0.861 Spearman’s rho and 0.761 Pearson’s coefficients). MLSA had a Pearson’s correlation coefficient of 0.829 with ANIm and a Spearman’s rho of 0.757 with ANI. The Spearman’s rho coefficients between *leu*S and ANI, ANIm and *leu*S, and TETRA and *leu*S were 0.842, 0.827, and 0.555, respectively (Supplementary Table S3). gDDH and ANIm had Pearson’s, Kendall’s tau and Spearman’s rho coefficients of 0.927, 0.880, and 0.825, respectively. The Pearson’s, Kendall’s tau and Spearman’s coefficients were 0.940, 0.882, and 0.478, respectively, between gDDH and ANI. ANI and ANIm had correlation coefficients of 0.911 (Spearman’s rho), 0.863 (Kendall’s tau), and 0.509 (Pearson’s coefficients). TETRA and ANI showed the lowest Pearson’s coefficients of 0.382 (Supplementary Table S3).

## Discussion

Members of the genus *Pantoea* are Gram-negative bacteria isolated from a various environments. The taxonomy and systematics of this varied group is not always clear. Initial classification of the species based on phenotypic or nutritional consistency as well as wet-lab DDH and 16S rRNA now requires revision with the advent of the rapid accumulation of genome data. The inconsistencies of the ‘gold standard’ wDDH values between laboratories suggest that, in some cases, strains might have been misclassified/identified. As the gold standard for bacterial classification shifts to whole genome-based indices, it is imperative to ascertain that genomes of available type and non-type strains are accurately identified to minimize errors in the systematics of this group. Using MLSA, ANI, ANIm, TETRA, and gDDH, Gomila et al. (2015) reported that 30% of the genomes of non-type strains were not correctly assigned at the species level within the *Pseudomonas* accepted taxonomical groupings. This could have significant implications on how the results of phylogenetic inference and identification are interpreted within the *Pseudomonas* genus in particular and the bacterial domain, in general. The declining number of classical bacterial taxonomists capable of species-level identification could exacerbate the problem of misclassification of bacteria. There is no report on the accuracy and validity of the constituted public genome sequence data for the genus *Pantoea*.

This is the first report analyzing 206 genomes for accurate assignment to species level within the genus *Pantoea*. This study used MLSA (cut-off threshold≥98%) and phylogenetic analysis, ANI (≥96%), ANIm (≥96%), TETRA (≥0.998), and gDDH (≥70%) data derived from each of the genome sequences to ascertain accurate classification. This system of five indices (MLSA and genome-based) was used to verify and assess the taxonomic status of 206 publicly available *Pantoea* genome sequences. Based on the respective cut-off threshold values for species delineation, about 11% of the genome sequences derived from non-type strains were found to be incorrectly assigned at the species-level within the genus *Pantoea*. For example, strain MHSD5 reported as a putative *P. ananatis* had similarity values below the species level cut-off threshold of MLSA, TETRA, ANI, ANIm, and gDDH with LMG 2665_T_, the type strain of *P. ananatis*. In contrast, the same strain had similarity values above the species-level cut-off threshold with strain LMG 24197T, the type strain of *P. eucalypti*, a clear indication that this strain is less taxonomically related to *P. ananatis*. Analysis of *Pseudomonas* genome sequences indicated that 30% of the non-type strains were not correctly assigned at the species-level (Gomila et al., 2015). This number is higher than what was found in the current study of genome sequences, suggesting a potentially widespread problem requiring some attention. Gomila et al. (2015) indicated that this could be due to the fact that the *Pseudomonas* strains were isolated and taxonomically classified using less reliable methods and as such their taxonomic status should be re-visited using modern techniques. Accurate species level identification of genome sequences is key to a better and reliable understanding of the bacterial phylogenomics, diversity and evolution. As the study of bacteria steadily shifts to genome analysis, accurate identification at the species level is primordial. It is crucial for the genus *Pantoea* given that its members are human and plant pathogens requiring the correct identity to identify effective management strategies. As such, the identification of “first-aid” markers to curate, rapidly and accurately, strains marked for genome sequencing can provide some assurance. This study is proposing a partial *leu*S gene fragment (642 bp), one of the MLSA genes designated as a reliable phylogenetic marker (Tambong et al., 2014), as the “first-aid” tool for the genus *Pantoea*. The 642-bp *leu*S fragment sequences correlated well with the species level assignations of MLSA and genome-based indices except TETRA patterns. This could be very helpful to scientist with limited experience in genome data analysis since conventional PCR and Sanger sequencing could be performed for this fragment and the strain identity verified by BLASTn targeting type strains of *Pantoea* species.

**FIGURE 6:**
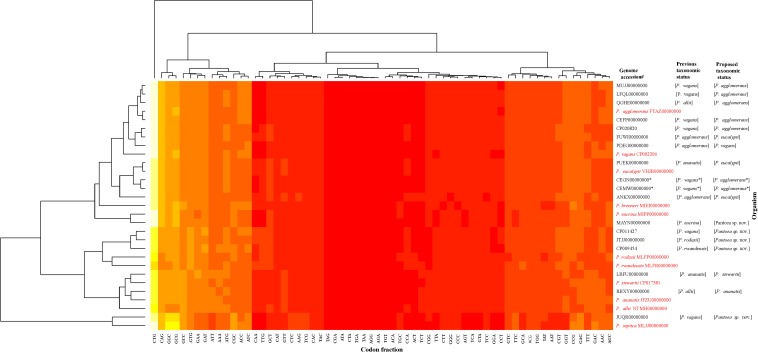
Codon usage pattern analysis of the species-level misidentified genome sequences relative to the type strains (in red) of the previous and new proposed taxonomic status. Heatmap was generated using a BioPerl module as implemented in CMG-Biotools pipeline (Vesth et al., 2013). Colors indicate discrepancy in codon usage among strains from low (yellow, <10%) to high occurrence (red, >85%). CEGN00000000 and CEMW00000000 genomes denoted by asterisk (^∗^) have almost double the size of expected genome of a *Pantoea* species and as such did not cluster with the MLSA-predicted relative, *P. agglomerans*.

The data generated from the indices were congruent in assigning most of the genome sequences at the species-level with the exception of two non-type strains (PaVv7 and PaVv9). The genome sequences of strains PaVv7 and PaVv9, initially reported to belong *to P. ananatis*, were transferred in this study to *P. agglomerans* based on indices derived from MLSA, ANI, and ANIm with values above the cut-off threshold. However, the gDDH value was 15.3% (species-level cut-off≥70%) and TETRA scores (cut-off≥0.998) of 0.994 (PaVv9,CEGN00000000) and 0.996 (PaVv7, CEMW00000000) between both strains and *P. agglomerans*, their new species level affiliation. This discrepancy between gDDH and TETRA compared to the other indices was validated by the results of codon usage patterns (Figure 6) that showed the two genomes clustering independently of the MLSA-predicted related species, *Pantoea agglomerans*. This could be attributed to the quality of the genome sequences. Firstly, each of these strains had a genome size of 9.8 Mb, almost twice the expected size of a typical *Pantoea* species. It is probable that the genome sequence was duplicated. This was evident during the extraction of the genes used in the MLSA analyses. These two genome sequences have duplicate and identical copies of the target gene fragments. All the other *Pantoea* genomes studied had single copies of *fu*sA, *gyr*B, *leu*S, *rpo*B, and *pyr*G genes. This is probably a mistake by the depositor(s) during the processing of the assembled data of strains PaVv7 and PaVv9. This affected gDDH values significantly, probably, because the first step of calculations is local alignment by BLAST to identify HSPs (Meier-Kolthoff et al., 2013). The duplicated genes in the genome sequences of strains PaVv9 and PaVv7 could affect the similarity scores required to compute the distance values. This did not affect the MLSA indices because the genes were extracted and manually curated before used.

This study analyzed 206 complete or draft genomes of *Pantoea* and found that 47 strains (23%) were not assigned to species (*Pantoea* sp.). A stringent process (all indices must exhibit values ≥ species level cut-off threshold) was used to ascertain correct assignation to a given validly published *Pantoea* species. Twenty-five genome sequences initially reported as *Pantoea* sp. were correctly assigned to 11 validly published *Pantoea* species. The same system assigned four strains to *Mixta calida* (formerly *Pantoea calida*). Seventeen of the *Pantoea* sp. strains, exhibited indices below the cut-off values relative to all the published *Pantoea* type strains; and based on this highly stringent system, these strains could be predicted to be putative novel species within the genus *Pantoea*. The genus level confirmation of these genomes was done based on 16S rRNA according to the similarity scores of 98.7–99% (Stackebrandt and Ebers, 2006) Also, one potential novel species was identified within the genus *Mixta* with *Mixta gavinae* (formerly *Pantoea gaviniae*) as the closest relative. These data were supported by the phylogenetic analysis based on MLSA. Polyphasic taxonomy (phenotypic, genotypic, and phylogenetic analyses) constitutes a milestone in modern bacterial taxonomy (Vandamme et al., 1996) but MLSA and genome analyses which include all the type strains can adequately predict putative novel species (Gomila et al., 2015). Currently, MLSA seems to be the method of choice for assessing the DNA relatedness within the genus *Pantoea* but as whole-genome sequences of the type strains become available there would be a shift to ANI and gDDH which have been demonstrated to be useful indices in species delineation. This is not surprising given that gDDH had the best correlation coefficient with MLSA.

In conclusion, improved NGS and computational systems are revolutionizing modern bacterial taxonomy. Genome sequence data from bacterial type strains can substantively improve our understanding of the extent and complexity of the phylogenetic space relative to previously reported taxonomic studies (Kyrpides et al., 2014). As whole-genomes of more bacterial type strains are sequenced, analyzed and made publicly available, this system could become the “new” gold standard. However, for reliability and accuracy, all genome sequences to be deposited in public databases should be curated by employing MLSA system specific to the target genus. For the genus *Pantoea*, the MLSA system and even the *leu*S “first-aid” tool described above could provide adequate data for an informed decision. Finally, incorrect species level assignation of strains can lead to fallacious conclusions especially in comparative genomics studies.

## Data Availability Statement

The datasets generated for this study can be accessed from the GenBank: VHJB00000000, VHIZ00000000, and VHJA00000000.

## Author Contributions

JT conceptualized the research, generated and analyzed the data, and wrote the manuscript.

## Conflict of Interest

The authors declare that the research was conducted in the absence of any commercial or financial relationships that could be construed as a potential conflict of interest.

## Footnotes

1 www.bacterio.net/pantoea.html

2 www.ncbi.nlm.nih.gov/genome/browser

## Supplementary Material

FIGURE S1 UGPMA Dendrogram generated using PermutMatrix showing two clusters: **(A)** genomes correctly identified as *P. vagans*, and **(B)** incorrect species-level assignment. 1, *leu*S; 2, MLSA; 3, Average nucleotide identity (ANI); 4, MuMmer-based ANIm; 5, Tetranucleotide patterns; and 6, genome-based DNA-DNA hybridization (gDDH). *P. vagans* C9-1 is used as a reference. ^∗^Genome sequences had two copies of each of the genes (double the size of average *Pantoea* genome) which might have affected the genome-based DDH values. ^∗∗^CP011427 could be a putative novel species with *Pantoea rodasii* as the closest relative.

TABLE S1 Strains, accession numbers, size and number of contigs of NCBI *Pantoea* genomes used in this study.

TABLE S2 Proposed species-level taxonomic affiliations based on 16S rRNA, *leu*S, concatenated MLSA and whole genome analyses of genome sequences previously reported as *Pantoea* sp.

TABLE S3 Pairwise correlation coefficients between *leu*S, MLSA, and genome-based data generated in this study.
