# Supplementary material for: Taxogenomics and Systematics of the Genus Pantoea
Source: Front Microbiol. 2019 Oct 30;10:2463. doi: 10.3389/fmicb.2019.02463 (PMC6831937; doi:10.3389/fmicb.2019.02463)
Supplement: TABLE S1 — Strains, accession numbers, size and number of contigs of NCBI Pantoea genomes used in this study. [file Data_Sheet_1.PDF]

Table S1. Strains, accession numbers, size and number of contigs of NCBI *Pantoea* genomes used in this study.

**Organism/Name**	**Strain code**	**WGS Accession number**	**Size (Mb)**	**Number of contigs**
Pantoea ananatis	LMG 2665T	JFZU00000000	4.98049	27
	LMG 2665	JMJJ00000000	4.9387	17
	LMG 20103	CP001875	4.70337	-
	PA13	CP003085	4.86713	-
	AJ13355	AP012032	4.87728	-
	LMG 5342	HE617160	4.90814	-
	R100	CP014207	4.85786	-
	YJ76	CP022427	5.14648	-
	97-1	CP020943	5.04741	3
	SGAir0210	CP028033	4.80869	2
	BRT175	ASJH00000000	4.85295	38
	Sd-1	AZTE00000000	4.92674	75
	BD442	JMJL00000000	4.79855	11
	PA4	JMJK00000000	5.16348	17
	CFH 7-1	LFLX00000000	4.59997	5
	S6	CVNF00000000	4.34478	77
	S7	CVNG00000000	4.49032	71
	S8	CVNH00000000	4.48322	61
	NS296	LDQX00000000	4.73486	54
	NS311	LDQZ00000000	4.72149	174
	RSA47	LDRA00000000	4.73791	46
	NS303	LDQY00000000	4.73297	69
	1.38	NKXT00000000	4.86928	23
	PANS 99-23	NMZS00000000	4.94101	59
	PANS 99-3	NMZR00000000	5.11382	54
	PANS 04-2	NMZV00000000	4.91688	62
	PANS 99-36	NMZT00000000	4.86262	68
	PANS 01-2	NMZU00000000	4.91923	45
	PNA 99-7	NMZW00000000	4.97176	65
	PNA 06-1	NMZY00000000	4.97769	58
	PNA 200-3	NMZX00000000	5.00881	66
	PNA 15-1	NMZZ00000000	4.99575	49
	MHSD5	PUEK00000000	4.65022	39
	PNA 14-1	QEKS00000000	5.052	39
	ARC311	PUGB00000000	4.59342	166
	ARC310	PUGC00000000	4.59824	216
	ARC272	PUGD00000000	4.61501	128
	B1-9	CAEI00000000	5.10556	169
	B1-9	CAEJ00000000	5.11924	233
	PaMB1	JQZZ00000000	4.7564	177
	AMG521	LMYG00000000	4.87577	79
	PNA 200-7	QGGN00000000	4.95166	21
	PNA 11-1	QGTK00000000	4.88607	30
	PNA 98-11	QGTO00000000	5.03718	40
	PNA 07-1	QICU00000000	4.90336	28
	PNA 86-1	QLSY00000000	4.82139	24
	PNA 07-10	QTTO00000000	4.89862	17
	PANS 02-01	QRDI00000000	4.99485	39
	PANS 200-1	QTTV00000000	4.8239	14

	NFR11	FPJM00000000	4.85463	16
	NFIX48	FUXY00000000	4.62424	16
	DAR76143	BATH00000000	5.24626	254
	MR5	LBFU00000000	4.14656	239
	UBA1895	DDGT00000000	4.77499	53
	UBA11896	DQCT00000000	3.46658	45
	UBA12293	DPYY00000000	4.71578	71
Pantoea agglomerans	DSM 3493T	FYAZ00000000	4.65938	33
	C410P1	CP016889	5.11524	-
	Tx10	ASJI00000000	4.85699	22
	Eh318	AXOF00000000	5.03584	34
	190	JNGC00000000	5.00257	5
	MP2	JPKQ00000000	4.73383	16
	JM1	NHAS00000000	4.79487	34
	Sl1_M5	ADWZ00000000	4.91973	37
	IG1	BAEF00000000	4.82958	18
	299R	ANKX00000000	4.58148	109
	RIT273	JFOK00000000	5.36534	26
	DAPP-PG734	JNVA00000000	5.36593	195
	LMAE-2	JWLQ00000000	4.98116	155
	GB1	JYGW00000000	4.76505	33
	P10c	LIME00000000	4.77592	18
	NBRC 102470	BCZA00000000	4.65204	25
	FDAARGOS_407	PDEG00000000	4.79547	5
	Bl3	QLAA00000000	5.04776	24
	BD 1212	QQXH00000000	4.8754	103
	BD 1274	QQXI00000000	4.96851	246
	NCTC10601	UGSL00000000	4.75619	6
	NCTC10500	UGSM00000000	4.65552	4
	4	JPOT00000000	4.82789	4
	3	LVHW00000000	4.81358	4
	824-1	LXSX00000000	4.98907	55
	4188	LXSW00000000	5.002	79
	P5	NGNU00000000	5.07426	127
	NFPP29	FUWI00000000	4.10462	13
	NCTC9381	UGSO00000000	4.69697	3
	UBA1585	DCNX00000000	4.57787	70
	UBA10729	DMFB00000000	2.26894	403
	UBA10742	DMXQ00000000	2.84373	542
	UBA8810	DOGI00000000	2.86453	450
Pantoea vagans	C9-1	CP002206	4.88834	
	ND02	CP011427	4.31326	
	FDAARGOS_160	CP014129	4.80886	
	PV989	CP028349	4.83915	
	FBS135	CP020820	4.94993	
	MP7	JPKP00000000	4.5987	8
	848_PVAG	JUQR00000000	4.99032	407
	ZBG6	LFQL00000000	4.729	92
	Pa	MUJJ00000000	4.79516	45
	TYU1	NRSO00000000	4.94223	6
	PaVv11	CEFP00000000	4.85077	84
	UBA6298	DJTW00000000	4.15881	299
	UBA6713	DKIR00000000	4.85705	25
	PaVv7	CEMW00000000	9.75451	385
	PaVv9	CEGN00000000	9.75451	385

Pantoea stewartii	DC283	CP017581	5.31409	-	
	DC283	AHIE00000000	5.23321	65	
	M009	JRWI00000000	4.8217	56	
	M073a	JSXF00000000	4.81761	39	
	A206	LIHC00000000	4.65461	19	
	NS381	LDSH00000000	4.693	51	
	RSA36	LDSK00000000	4.79039	76	
	RSA30	LDSJ00000000	4.76037	84	
	RSA13	LDSI00000000	4.7665	48	
	S301	LIIU00000000	4.48641	27	
	LMG 2632T	JPKO00000000	4.68124	35	
Pantoea dispersa	NS380	LDSA00000000	4.80103	123	
	EGD-AAK13	AVSS00000000	4.76458	39	
	NS375	LDRZ00000000	4.88722	42	
	SA3	LDSE00000000	4.90522	83	
	NS215	LDRY00000000	4.84387	35	
	NS389	LDSB00000000	4.81979	54	
	SA2	LDSD00000000	4.90615	52	
	SA5	LDSG00000000	4.89702	98	
	RSA31	LDSC00000000	4.94953	194	
	SA4	LDSF00000000	4.91108	58	
	SUBG008	LEKX00000000	4.44488	8097	
	UBA3148	DEYW00000000	4.6657	35	
Pantoea septica	LMG 5345T	MLJJ00000000	4.30872	89	
	FF5	CCAQ00000000	4.54808	37	
	UBA1650	DCLK00000000	3.8387	243	
	UBA2053	DDAR00000000	3.17082	313	
	UBA2049	DDAV00000000	4.19715	40	
	UBA2584	DDLA00000000	4.31382	34	
	UBA1889	DDGZ00000000	3.71122	361	
	UBA7459	DLPL00000000	4.02392	132	
	UBA11341	DMYN00000000	2.46265	422	
Pantoea allii	LMG 24248	NTMH00000000	5.24114	57	
	PNA 200-10	QGHF00000000	5.00087	26	
	PNG 92-11	QGHE00000000	5.05806	36	
	PNA 02-18	RBXY00000000	4.85908	19	
	LMG 24248	MLFE00000000	5.01068	92	
Pantoea eucrina	LMG 5346T	MIPP00000000	3.89238	109	-
	Russ	MAYN00000000	3.93988	8	-
Pantoea brenneri	LMG 5343	MIEI00000000	4.93318	241	
	IF5SW-P1	MIZY00000000	5.12279	276	
Pantoea rodasii	LMG 26273	MLFP00000000	5.75146	86	
	ND03	JTJJ00000000	5.68029	213	
	DSM 26611	PIQI00000000	5.74806	31	
Pantoea rwandensis	LMG 26275	MLFR00000000	5.7754	69	
	ND04	CP009454	4.32761	-	
Pantoea alhagi	LTYR-11Z	CP019706	4.24	-	
Pantoea anthophila	11-2	JXXL00000000	4.6	16	
Pantoea coffeiphila	342	PDET00000000	5.88	93	
Pantoea conspicua	LMG 24534T	MLFN00000000	4.31	128	
Pantoea cypripedii	LMG 2657T	MLJI00000000	6.55	3	
Pantoea deleyi	LMG 24200T	MIPO00000000	4.61	361	
Pantoea latae	AS1T	MWUE00000000	4.96	83	
Pantoea sesami	Si-M154	FQWJ00000000	4.91	35	
Pantoea wallisii	LMG 26277	MLFS00000000	4.65	149	

Pantoea sp.	At-9b	CP002433	6.31278	-
	PSNIH1	CP009880	4.41068	-
	PSNIH2	CP009866	4.96734	-
	aB	AEDL00000000	4.80531	33
	Sc1	AJFP00000000	4.44721	4
	GM01	AKIU00000000	5.32055	102
	YR343	AKIT00000000	5.31405	128
	A4	ALXE00000000	5.32972	71
	AS-PWVM4	ASZC00000000	4.99087	46
	IMH	JFGT00000000	4.09115	8
	3.5.1	JMRT00000000	4.96465	23
	NGS-ED-1003	JPQA00000000	4.80906	49
	9140	JQNO00000000	4.83947	3
	MBLJ3	JSUT00000000	4.82132	37
	BL1	JZRH00000000	5.33146	121
	SM3	JZRD00000000	5.33219	89
	RIT-PI-b	LGIS00000000	5.27169	35
	CFSAN033090	LGYX00000000	4.94297	47
	1.19	MRBS00000000	3.79274	41
	VS1	NHZE00000000	5.41283	191
	AMG 501	NIRH00000000	5.10264	51
	AV62	NHBE00000000	4.97337	57
	FDAARGOS_194	NWFM00000000	4.87714	6
	596	PJRT00000000	5.1145	33
	PSNIH5	PQJX00000000	4.93717	84
	PSNIH4	PQJZ00000000	4.95105	87
	PSNIH6	PQJV00000000	5.35113	70
	PSNIH3	PQJW00000000	4.93104	106
	ICBG 1758	POWL00000000	4.17552	35
	ICBG 828	POWN00000000	5.02371	35
	ICBG 985	POWM00000000	4.93163	33
	RIT 413	QBJB00000000	3.83688	38
	JKS000250	QICZ00000000	4.86852	3
	AG1095	QKMJ00000000	4.91479	25
	ARC607	PUFZ00000000	4.40532	190
	ARC270	PUGA00000000	4.39716	91
	3_1284	QNVM00000000	4.07209	67
	OXWO6B1	LWLR00000000	5.23614	5
	Ae16	MDJQ00000000	3.99662	41
	BRM17	PEFU00000000	3.27324	3
	AG702	QGTU00000000	4.75273	23
	PNA 03-3	QICO00000000	4.93289	22
	YR512	FOSD00000000	5.27708	27
	OV426	FOVG00000000	4.75126	12
	YR525	FPBU00000000	5.27687	27
	GL120224-02	OBEC00000000	5.11881	27
	B40	BADG00000000	4.64523	728

-, complete genomes
