# Supplementary material for: Taxogenomics and Systematics of the Genus Pantoea
Source: Front Microbiol. 2019 Oct 30;10:2463. doi: 10.3389/fmicb.2019.02463 (PMC6831937; doi:10.3389/fmicb.2019.02463)
Supplement: TABLE S2 — Proposed species-level taxonomic affiliations based on 16S rRNA, leuS, concatenated MLSA and whole genome analyses of genome sequences previously reported as Pantoea sp. [file Data_Sheet_2.PDF]

Table S2. Proposed species-level taxonomic affiliations based on 16S rRNA, *leu* S, concatenated MLSA and whole genome analyses of genome sequences previously reported as *Pantoea sp* .[1]

Organism	Strain code	Genome Accession #	*leu* S (%)	MLSA (%)	gDDH (%)	ANI (%)	ANIm (%)	TETRA	Proposed species-level identification[2]	Genome accession # of the closest species
Pantoea sp.	CFSAN033090	LGYX00000000	99.00	99.00	88.70	98.45	98.64	0.9996	*P. agglomerans*	FYAZ00000000
	OXWO6B1	LWLR00000000	99.00	99.00	89.70	98.58	98.85	0.9994	*P. allii*	NTMH00000000
	AMG 501	NIRH00000000	99.00	99.00	90.90	98.40	99.04	0.9994	*P. ananatis*	JFZU00000000
	AG702	QGTU00000000	99.00	99.00	93.20	98.96	99.19	0.9996	*P. ananatis*	
	B40	BADG00000000	99.00	99.00	92.70	98.81	99.09	0.9996	*P. ananatis*	
	Sc1	AJFP00000000	98.75	97.00	87.30	98.26	98.54	0.9997	*P. anthophila*	This study
	ARC270	PUGA00000000	98.75	97.00	87.30	98.30	98.54	0.9998	*P. anthophila*	
	AV62	NHBE00000000	99.75	99.00	99.80	99.94	99.96	0.9998	*Pantoea brenneri*	MIEI00000000
	3.5.1	JMRT00000000	99.32	99.00	95.50	99.30	99.44	0.9997	*P. brenneri*	
	VS1	NHZE00000000	98.75	99.00	83.10	97.86	98.13	0.9989	*P. dispersa*	LDSA00000000
	ICBG 828	POWN00000000	99.00	99.00	84.10	98.04	98.24	0.9997	*P. dispersa*	
	ICBG 985	POWM00000000	99.00	99.00	84.70	98.08	98.28	0.9997	*P. dispersa*	
	aB	AEDL00000000	99.00	99.00	91.80	98.96	99.06	0.9998	*P. eucalypti*	This study
	MBLJ3	JSUT00000000	98.00	99.00	92.80	99.05	99.13	0.9998	*P. eucalypti*	
	3_1284	QNVM00000000	99.00	99.00	97.40	99.52	99.65	0.9997	*P. eucrina*	MIPP00000000
	Ae16	MDJQ00000000	99.00	99.00	90.00	98.65	98.89	0.9996	*P. eucrina*	
	RIT 413	QBJB00000000	99.00	99.00	91.00	98.82	98.99	0.9997	*P. eucrina*	
	PSNIH1	CP009880	99.00	99.00	89.90	98.63	98.90	0.9985	*P. eucrina*	
	BRM17	PEFU00000000	99.00	99.00	90.10	98.13	98.92	0.9988	*P. eucrina*	
	ICBG 1758	POWL00000000	99.00	99.00	91.60	98.77	99.03	0.9996	*P. eucrina*	
	FDAARGOS_194	NWFM00000000	98.00	98.00	67.70	95.93	96.17	0.9990	*Pantoea septica*	MLJJ00000000
	NGS-ED-1003	JPQA00000000	98.00	98.00	67.00	95.85	96.04	0.9988	*P. septica*	
	PNA 03-3	QICO00000000	99.00	99.00	90.40	98.77	99.13	0.9942	*P. s. subsp. stewartii*	CP017581
	9140	JQNO00000000	98.00	99.00	88.30	98.46	98.67	0.9989	*Pantoea vagans*	CP002206
	JKS000250	QICZ00000000	98.00	99.00	87.80	98.48	98.61	0.9990	*P. vagans*	
Pantoea sp. nov.	OV426	FOVG00000000	94.00	91.00	34.20	88.17	88.52	0.9844	*Pantoea sp.* nov. (*P. anthophila*)	This study
	**ARC607**	PUFZ00000000	99.00	99.00	58.10	94.69	94.80	0.9972	*Pantoea sp.* nov. *(P. deleyi)*	This study
	AS-PWVM4	ASZC00000000	85.00	91.00	31.30	86.87	87.14	0.9891	*Pantoea sp.* nov. *(P. cypripedii)*	MLJI00000000
	At-9b	CP002433	85.36	91.00	32.00	87.49	87.38	0.9891	*Pantoea sp.* nov. (*P. cypripedii*)	
	A4	ALXE00000000	83.00	87.00	21.30	80.35	83.91	0.8633	*Pantoea sp.* nov. (*P. dispersa*)	LDSA00000000
	AG1095	QKMJ00000000	87.00	92.00	27.50	84.91	85.52	0.9701	*Pantoea sp.* nov. (*P.rodasii)*	MLFP00000000
	GM01	AKIU00000000	84.00	91.00	27.30	84.73	85.45	0.9802	*Pantoea sp.* nov. (*P. rodasii*)	
	596	PJRT00000000	87.00	91.00	27.50	84.89	85.53	0.9702	*Pantoea sp.* nov. (*P. rodasii*)	
	YR343	AKIT00000000	85.00	91.00	27.60	85.20	85.56	0.9682	*Pantoea sp.* nov. (*P. rodasii*)	
	YR525	FPBU00000000	85.00	93.00	27.60	85.11	85.56	0.9677	*Pantoea sp.* nov. (*P. rodasii*)	
	GL120224-02	OBEC00000000	91.88	93.00	36.70	89.42	89.29	0.9961	*Pantoea sp.* nov. (*P. rodasii*)	
	BL1	JZRH00000000	93.00	93.00	35.60	88.98	88.93	0.9955	*Pantoea sp.* nov. (*P. rodasii*)	
	RIT-PI-b	LGIS00000000	85.00	91.00	27.20	84.63	85.42	0.9783	*Pantoea sp.* nov. (*P. rodasii*)	
	YR512	FOSD00000000	85.00	93.00	27.60	85.07	85.56	0.9677	*Pantoea sp.* nov. *(P. rodasii)*	
	SM3	JZRD00000000	93.00	93.00	31.40	87.22	87.49	0.9825	*Pantoea sp.* nov. (*P. rwandensis*)	MLFR00000000
	IMH	JFGT00000000	85.00	885.00	20.80	79.21	83.87	0.8310	*Pantoea sp* . nov. *(P. septica)*	MLJJ00000000
	PSNIH6	PQJV00000000	94.00	96.00	49.60	92.68	93.00	0.9932	*Pantoea sp.* nov. *(P. septica)*	
Mixta sp.	*PSNIH2*	CP009866	99.00	99.00	97.10	99.55	99.62	0.9997	*P. calida* /*Mixta calida*	CP026378
	PSNIH3	PQJW00000000	99.00	99.00	97.00	99.51	99.60	0.9977	*Mixta calida*	CP026378
	PSNIH5	PQJX00000000	99.00	99.00	97.00	99.47	99.59	0.9976	*Mixta calida*	
	PSNIH4	PQJZ00000000	99.00	99.00	97.00	99.51	99.59	0.9976	*M. calida*	
	1.19	MRBS00000000	85.00	87.00	21.30	79.47	84.12	0.9159	*Mixta sp.* nov *(Mixta gaviniae)*	CP026377

[1] MultiLocus Sequence Analysis (MLSA; species cut-off =97%) was done by concatenation of *fus* A, *gyr* B, *leu* S, *pyr* G and *rpo* B as described by Tambong *et al* (2014). Genome-based DNA-DNA hybridization (gDDH; species cut-off=70%) was calculated as decsribed by Meier-Kolthoff et al. (2013); average nucleotide identity (ANI; species cut-off =96%) calculated using FASTANI (Jain et al., 2018); MuMmer-based ANI (ANIm; cut-off =96%) and Tetranucleotide values (TETRA; species cut-off =0.998) were computed using JSpecies (Richter M, Rosselló-Móra, 209). [2]Species in brackets are the closest *Pantoea* relatives of proposed novel taxa. n/a, not available.
