# Supplementary material for: Taxogenomics and Systematics of the Genus Pantoea
Source: Front Microbiol. 2019 Oct 30;10:2463. doi: 10.3389/fmicb.2019.02463 (PMC6831937; doi:10.3389/fmicb.2019.02463)
Supplement: TABLE S3 — Pairwise correlation coefficients between leuS, MLSA, and genome-based data generated in this study. [file Data_Sheet_3.PDF]

Table S3. Pairwise correlation coefficients between leuS, MLSA and genome-based data generated in this study*.

	Correlation index	leuS	MLSA	ANI	ANIm	TETRA	GGDC
leu S	Pearson	1	0.922	0.525	0.861	0.629	0.897
	Kendall's tau	1	0.728	0.730	0.721	0.450	0.731
	Spearman's rho	1	0.79	0.842	0.827	0.555	0.843
MLSA	Pearson	0.923	1	0.509	0.829	0.661	0.861
	Kendall's tau	0.728	1	0.647	0.636	0.503	0.646
	Spearman's rho	0.790	1	0.757	0.741	0.606	0.761
ANI	Pearson	0.525	0.509	1	0.466	0.382	0.478
	Kendall's tau	0.730	0.647	1	0.863	0.423	0.882
	Spearman's rho	0.842	0.757	1	0.911	0.555	0.400
ANIm	Pearson	0.861	0.829	0.466	1	0.577	0.825
	Kendall's tau	0.721	0.636	0.863	1	0.462	0.880
	Spearman's rho	0.827	0.741	0.911	1	0.608	0.927
TETRA	Pearson	0.629	0.661	0.382	0.577	1	0.512
	Kendall's tau	0.450	0.503	0.424	0.462	1	0.422
	Spearman's rho	0.555	0.606	0.555	0.608	1	0.560
GGDC	Pearson	0.843	0.761	0.940	0.927	0.561	1
	Kendall's tau	0.731	0.646	0.882	0.880	0.422	1
	Spearman's rho	0.897	0.861	0.478	0.825	0.512	1

* All correlation coefficients are significant at p=0.00. Correlation coefficents were computed in R-statistics (reference) using the cor function (Langfelder and Horvath, 2012)
