# Supplementary material for: Taxogenomics and Systematics of the Genus Pantoea
Source: Front Microbiol. 2019 Oct 30;10:2463. doi: 10.3389/fmicb.2019.02463 (PMC6831937; doi:10.3389/fmicb.2019.02463)

Tambong (2019) Frontiers in Microbiolol. XX:xx-xx

Figure S1

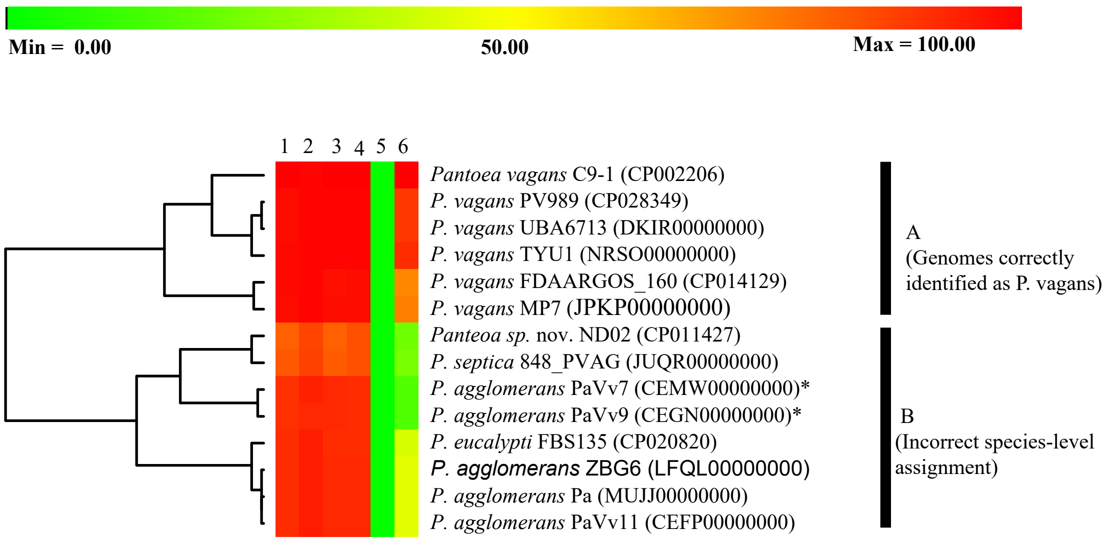

Supplement: FIGURE S1 — UGPMA Dendrogram generated using PermutMatrix showing two clusters: (A) genomes correctly identified as P. vagans, and (B) incorrect species-level assignment. 1, leuS; 2, MLSA; 3, Average nucleotide identity (ANI); 4, MuMmer-based ANIm; 5, Tetranucleotide patterns; and 6, genome-based DNA-DNA hybridization (gDDH). P. vagans C9-1 is used as a reference. ∗Genome sequences had two copies of each of the genes (double the size of average Pantoea genome) which might have affected the genome-based DDH values. ∗∗CP011427 could be a putative novel species with Pantoea rodasii as the closest relative. [file Data_Sheet_4.pdf]
